# ‘On Your Feet to Earn Your Seat’: update to randomised controlled trial protocol

**DOI:** 10.1186/s13063-015-0868-x

**Published:** 2015-08-05

**Authors:** Benjamin Gardner, Lee Smith, Daniel Aggio, Steve Iliffe, Kenneth R. Fox, Barbara J. Jefferis, Mark Hamer

**Affiliations:** Department of Psychology, Institute of Psychiatry, Psychology and Neuroscience (IoPPN), King’s College London, London, UK; UCL Centre for Behaviour Change, University College London, London, UK; Health Behaviour Research Centre, Department of Epidemiology and Public Health, University College London, London, UK; Research Department of Primary Care & Population Health, University College London, London, UK; Centre for Exercise, Nutrition and Health Sciences, University of Bristol, Bristol, UK; Population Health Domain Physical Activity Research Group, Department of Epidemiology and Public Health, University College London, London, UK

**Keywords:** Sedentary behaviour, Physical activity, Behaviour change, Habit, Older adults

## Abstract

**Background:**

This update describes changes to procedures for our randomised controlled trial of ‘On Your Feet to Earn Your Seat’, a habit-based intervention to reduce sedentary behaviour in older adults. Some of the amendments have arisen from the addition of new sites, each offering different possibilities and constraints for study procedures. Others have been made in response to problems encountered in administering intended recruitment procedures at the London sites described in our original protocol. All changes have received ethics and governance clearance, and were made before or during data collection and prior to analyses.

**Methods/design:**

Five non-London UK NHS-based sites (three general practices, one hospital, one NHS Foundation Trust) have been added to the study, each employing locally-tailored variations of recruitment and data collection procedures followed at the London sites. In contrast to the London sites, accelerometry data are not being collected nor are shopping vouchers being given to participants at the new sites. Data collection was delayed at the London sites because of technical difficulties in contacting participants. Subsequently, a below-target sample size was achieved at the London sites (*n* = 23), and recruitment rates cannot be estimated. Additionally, the physical inactivity inclusion criterion (i.e., <30 consecutive minutes of leisure time activity) has been removed from all sites, because we found that participants at the London sites meeting this criterion at consent subsequently reported activity above this threshold at the baseline assessment.

**Conclusion:**

This is primarily a feasibility trial. The addition of new sites, each employing different study procedures, offers the opportunity to assess the feasibility of alternative recruitment and data collection methods, so enriching the informational value of our analyses of primary outcomes. Recruitment has finished, and the coincidence of a small sample at the London sites with addition of new sites has ensured a final sample size similar to our original target.

**Trial registration:**

Current Controlled Trials ISRCTN47901994 (registration date: 16th January 2014)

## Update

This update describes changes to the recruitment and data collection procedures used in the ongoing randomised controlled trial of our habit-based intervention to reduce sedentary behaviour in older adults (titled ‘On Your Feet to Earn Your Seat’). This update should be read in conjunction with our original study protocol [1]. Most of the amendments detailed below arose in response to two developments: expansion of the study to new sites, and difficulties experienced in administering intended recruitment procedures at the London sites.

### Additional study sites

In accordance with standard UK National Health Service ethics and governance procedures, the study was advertised on the UK Clinical Research Network Database. A number of NHS sites in England approached the research team to request to join the study. Sites were accepted for the study only where they had sufficient resources to undertake recruitment and data collection independently of the research team. Five new sites were added. Study procedures were tailored according to the practical constraints of each site, following discussions between the Chief Investigator (BG) and local investigators.

### Recruitment difficulties at London sites

Following a patient record search, potentially eligible patients were sent a study information pack, including a business reply envelope for returning expressions of interest to the research team. A commercial mailout company sent the first batch of information packs to 300 patients of one general practice in August 2014. Only seven expressions of interest were received within three weeks. We discovered that an unknown number of these information packs contained an envelope with the incorrect reply address, and some did not contain a reply envelope. The mailout was reissued, alongside a new batch from a second general practice, in September 2014. Additionally, most reply envelopes received by the research team were marked ‘unpaid’ by the postal service. The postal service advised that this was an error, but that it was possible that some expressions of interest may not have been delivered. These errors delayed recruitment, such that only 23 participants could be recruited at the London sites because of finite time, budget, and staffing resources.

## Revised trial procedures

All procedures remain as detailed in our original protocol [1], with the following exceptions.

### Study setting

Participants were recruited through one of seven sites: two general practices from London (‘London sites’), as in our published protocol [1]; an NHS Trust in Lincolnshire, north England (‘Lincs site’); an outpatients clinic from a District General Hospital in Surrey, south England, specialising in Care of the Elderly (‘Surrey site’); and three general practices in Kent, south England (‘Kent sites’). Each site is led by a Clinical Studies Officer and has a history of research activity. Participants at all sites provided written informed consent prior to enrolment in the study.

**Fig 1:**
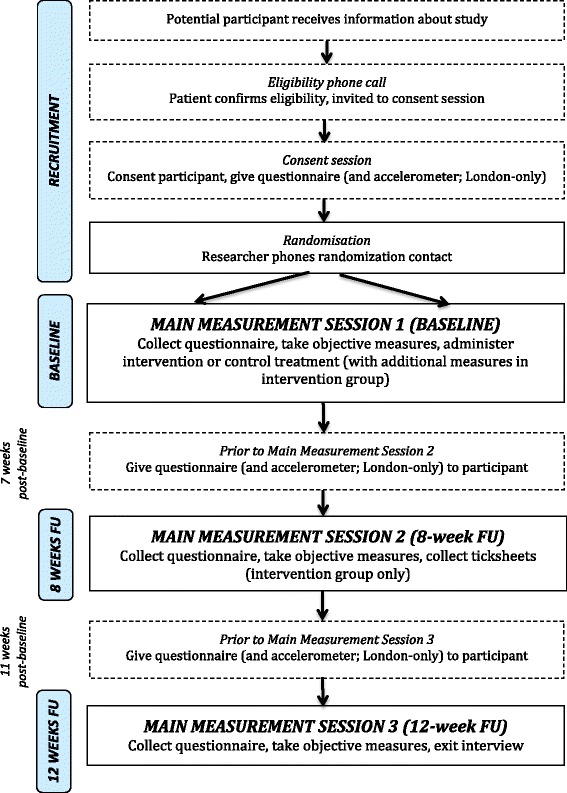
Participant flow, all sites

Recruitment at all sites ended in January 2015. Each site began recruitment at different times. Recruitment and data collection procedures vary between each sites. Key differences are summarised in Table 1 and below. Figure 1 illustrates the recruitment and data collection procedures common to all sites.

**Table 1:** Sources of variation in study procedures across research sites

	London	Lincs	Surrey	Kent
Recruitment				
Host site	General practices x 2	Foundation trust	General hospital (outpatients)	General practices x 3
Identification of participants	Direct mailout	Public advertisements	Direct mailout	Direct mailout
Data collection				
Data collection sites	Participant’s home	Participant’s home	Research clinic	Research clinic or participant’s home
Objective health and wellbeing variables	Walking speed	Walking speed	Walking speed	Walking speed
	Grip strength	Leg strength	Leg strength	Leg strength
	Leg strength	Balance	Balance	Balance
	Balance	Blood pressure	Blood pressure	Blood pressure
	Blood pressure			
Physical activity and sedentary behaviour measures	Objective + self-report	Self-report	Self-report	Self-report
Shopping vouchers given	Yes	No	No	No

At London and Lincs sites, data are collected in participants’ homes. At the Surrey site, data are collected at a research clinic in the host hospital. At the Kent sites, data are collected either in a research clinic or in the participant’s home, according to participant preference.

Researchers at each site were fully trained in recruitment, data collection and intervention and control administration by the Chief Investigator, and received a site-specific manual detailing all localised trial procedures.

### Inclusion criteria: participants

Our original inclusion criteria required that participants were self-reportedly aged 60 to 74 years, retired, physically inactive (i.e., ≤30 consecutive minutes of leisure time physical activity of ≥3 metabolic equivalents per week) and sedentary (≥6 total leisure time hours sitting per day). Early experiences at the London sites suggested that those self-reportedly meeting the inactivity criterion at consent had increased their activity at baseline (one week later), such that they no longer met the criterion. Sedentary behaviour is thought to have a detrimental health impact even when controlling for physical activity [2, 3], and so it is possible that any sedentary older person may benefit from our intervention or the control treatment, both of which outline the importance of reducing sitting time [1]. While we continued to seek less active older adults for the study, the inactivity eligibility criterion was removed, to maximise data availability.

### Recruitment procedure: participants

#### Lincs site

Posters were displayed in GP surgeries and community settings, and on local radio, advertising the study and inviting older adults aged 60–74 years who ‘do not do much physical activity’ to participate, and providing contact details for the local team. Interested potential participants who phoned the local research team were given an explanation of the study purpose. Their eligibility was checked and contact details recorded, and they were subsequently mailed a study information pack. One week later, a local researcher phoned respondents to confirm interest in the study and invite them to the consent session. Subsequent recruitment procedures were as stated in our original protocol [1].

#### Surrey site

Participants were identified from records of current and past outpatients, as screened by a local researcher. Patients who met our exclusion criteria were removed; all others were eligible for contact by the research team. Subsequent recruitment procedures were as stated in our original protocol [1].

#### Kent sites

Recruitment followed the same procedures as stated in our original protocol [1], except that expressions of interest from potential participants were returned to the London research team (in the same reply envelopes that had caused difficulty at the London sites), electronically scanned and sent to local investigators at a secure email address.

### Randomization

Site-specific random number lists, generated by a trial administrator at University College London, were used to achieve a 1:1 allocation ratio at each site. The administrator randomized participants at all sites via phone.

### Study procedures

We originally intended to fit each participant with an activPAL accelerometer, and recognise their contribution by giving them shopping vouchers. Funding at the non-London sites covers staffing costs only, and so neither accelerometers nor shopping vouchers have been used among participants at Lincs, Surrey, or Kent sites. Non-London sites were provided, on request, with blood pressure monitors and stopwatches to collect objective functioning data, and digital recorders to capture interview data. There were insufficient funds to provide hand dynamometers, for grip strength measurement, to non-London sites.

### Analysis plan

#### Analysis of primary outcomes

Recruitment rates will not be reported because they cannot be estimated at London or Kent sites due to mailout problems, nor at the Lincs site because the number of potential participants exposed to study advertisements is not known. Our revised analyses of primary outcomes will focus on describing the duration of measurement visits, rates of attrition and adherence to tips, and a thematic analysis of participants’ views towards study procedures and reasons for participating, as expressed in qualitative interviews. Due to limited resources, only five qualitative interviews from each of the four site clusters (London, Lincs, Surrey, Kent), identified using a random number generator, will be selected for transcription and analysis.

#### Analysis of secondary outcomes

Exploratory analyses of changes in physical activity, habit, wellbeing, physical health, functioning (secondary outcomes) will proceed as detailed in our original protocol, with the exception that objective physical activity and sedentary behaviour data analysis will only be possible for participants at the London sites, for whom accelerometry data are available. Where possible, self-reported activity and sitting will be verified against accelerometry data. No adjustments will be made to statistical analyses to account for clustering effects, because the study will likely be insufficiently powered for multi-level modelling based on seven recruitment sites.

### Ethics

The changes detailed in this update have been approved as a series of Substantial and Non-Substantial Amendments by the Bromley NHS Research Ethics Committee (reference 13/LO/1549). Site-specific governance approvals were provided by North Thames Clinical Research Network (CRN; London sites), East Midlands CRN (Lincs site), and Kent, Surrey and Sussex CRN (Surrey and Kent sites). The independent project steering committee was notified of all changes and no objections were raised.

## Conclusion

We have reported major amendments made to our ongoing study, so as to increase transparency and justify discrepancies between our intended and actual procedures, ahead of dissemination of findings. The study is due to end in April 2015 - recruitment has been completed, but data collection is ongoing - and so we do not anticipate any further changes. These amendments will enrich the informational value of the study. We aimed to recruit 120 older adults to the London sites, but recruitment difficulties resulted in only 23 participants being consented. However, the coincidental expansion of the study to new sites has largely mitigated recruitment problems, such that our final baseline sample is similar to our original target (109 participants have been consented at all sites).

This is primarily a feasibility trial. The addition of new sites, each employing locally-tailored recruitment and data collection procedures, offers the opportunity to assess multiple alternative procedures as part of our analyses of primary outcomes. This will allow us to determine the optimal strategies for a future definitive RCT. For example, future trial costs will be lowered should we find that participants at the non-London sites respond positively to study procedures despite not receiving shopping vouchers. The main impact of the changes to be on analyses of secondary outcomes; that is, changes in behaviour, habit, health and wellbeing. Objective activity and sitting data will only be available for participants at the London sites, with whom activPAL accelerometers are being used to collect postural allocation data that detect true activity patterns more reliably than self-report [4, 5]. The sensitivity of self-reported physical activity to true intervention-related changes has also been questioned [6], such that potential intervention effects may go undetected. However, we were aware of these limitations when we selected changes in self-reported physical activity as the outcome for which effect size estimates would be derived for the purpose of powering a future large-scale trial [1]. The availability of accelerometer data from at least some sites means that the accuracy of self-reported activity (and sitting) can be explored among a sub-sample of participants. Similarly, we acknowledge that analyses of changes in behaviour, habit, health and wellbeing may not be conclusive, because our total sample size is likely to be too small to adequately power multi-level models that can account for clustering effects within each of the seven sites. However, we did not originally intend to correct for clustering within the two recruitment sites [1], because our analyses of secondary outcomes are exploratory rather than definitive. The main aim of this trial is to assess whether the intervention should be recommended for more rigorous tests of effectiveness, and to what extent our study procedures are fit for this purpose. We do not therefore view the changes we have detailed here to be problematic.

## Trial status

The trial is ongoing. Recruitment has been completed. Data collection is ongoing. The trial will end on 30th April 2015.

## Abbreviations

CRN: Clinical Research Network
NHS: National Health Service.
